# Personalization of Minimally-Invasive Aesthetic Procedures with the Use of Ultrasound Compared to Alternative Imaging Modalities

**DOI:** 10.3390/diagnostics13233512

**Published:** 2023-11-23

**Authors:** Cristina Beiu, Liliana Gabriela Popa, Beatrice Bălăceanu-Gurău, Carmen Andrada Iliescu, Andreea Racoviță, Marius Nicolae Popescu, Mara Mădălina Mihai

**Affiliations:** 1 Department of Oncologic Dermatology, “Elias” Emergency University Hospital, “Carol Davila” University of Medicine and Pharmacy, 020021 Bucharest, Romania; cristina.beiu@umfcd.ro (C.B.); liliana.popa@umfcd.ro (L.G.P.); mara.mihai@umfcd.ro (M.M.M.); 2 Clinic of Dermatology, “Elias” Emergency University Hospital, 011461 Bucharest, Romania; andreea.stefania.racovita@gmail.com; 3 Department of Physical and Rehabilitation Medicine, “Elias” Emergency University Hospital, “Carol Davila” University of Medicine and Pharmacy, 020021 Bucharest, Romania; marius.popescu@umfcd.ro; 4 Clinic of Physical and Rehabilitation Medicine, “Elias” Emergency University Hospital, 011461 Bucharest, Romania

**Keywords:** high-frequency ultrasound, hyaluronic acid fillers, aesthetic procedures, biostimulators, injectables complications

## Abstract

Ultrasonography is a well-tolerated procedure that aids in the targeted esthetic therapies of special cutaneous regions, as well as in the prevention (vascular mapping, identification of previous filler, and others) and management of potential complications (vascular occlusion, external vascular compression, product misplacement or migration, inflammatory reactions, and others). It has lately been promoted as the first-line imaging tool to address injectables. In this article, we aim to review the evolving role of ultrasonography in cosmetic filler procedures, from the fundamental ultrasound characterization of cosmetic fillers to the ultrasound-enhanced detection and management of cosmetic filler complications, including ultrasound’s role in hyaluronidase-guided injections for cosmetic filler dissolution. Furthermore, the paper explores the integral role played by ultrasound in enhancing the precision, efficacy, and safety of additional minimally invasive aesthetic techniques such as mesotherapy, radiofrequency, cryolipolysis, and polydioxanone procedures.

## 1. Introduction

Ultrasound (US) has gained widespread recognition as a valuable tool in skin imaging over the course of recent years. Being first used in clinical dermatology in 1979, it has expanded beyond its traditional boundaries, now serving as an ancillary tool to assist dermatologists in diagnostic and therapeutic procedures [1]. It is a non-irradiating and highly accessible imaging modality that enables not only the observation of all skin layers, appendages, and deeper structures, but also the estimation of vascular anatomy using Doppler mode [2]. US is used for the diagnosis of both benign and malignant skin tumors, inflammatory dermatoses, and nail and scalp pathology, as well as for the evaluation of foreign bodies and aesthetic fillers [3].

In the fields of cosmetology and medical aesthetics, high-resolution US technology equipped with probes in the upper-frequency range, between 15 and22 MHz, is the perfect tool to provide objective information before, during, and after different aesthetic procedures such as volumetric aesthetic enhancement treatments or other minimally invasive rejuvenating therapies [4,5].

The use of filler materials for aesthetic enhancement has become more and more common over the years, with a large selection of products being accessible for both certified medical personnel and illegally operating ones. Patients are therefore exposed to a wide range of complications, greater if non-medical approved substances are utilized [6]. Moreover, an increasing number of patients have an unclear record of injections, making it difficult to assess the type of product used or to link an adverse reaction to it [7]. For all these situations, US imaging is the ideal method to determine the type of filler utilized based on the different sonographic patterns of echogenicity and posterior acoustic artifacts [8]. It can also provide further information about the precise location of volumetric substances, previous implants, and their potential interactions with surrounding tissues [9]. Furthermore, sonography in Duplex mode can be of great value in improving the safety profile of filler treatments [10]. Vascular mapping can be performed before and after aesthetic procedures, especially in the high-risk regions of the face to prevent undesired adverse events such as tissue necrosis or ischemia. If vascular occlusion or external compression due to hyaluronic acid injection occurs, targeted vessels can be identified and US-guided application of hyaluronidase can be performed to reestablish the vascular flow [11]. US is also helpful in providing early detection and improved management of many other filler-related complications, such as inflammatory reactions, filler migration and displacement, allergic reactions, or infections [2]. 

Beyond filler materials, US is also gaining popularity in other minimally invasive aesthetic techniques such as mesotherapy, radiofrequency, and cryolipolysis, as there was much need for a non-invasive and easy-to-recreate method to objectively quantify the effects and progress of these prominent and extensively employed procedures [12,13,14]. Several parameters, such as the thickness of the epidermis and dermis, echogenicity of skin layers, level of swelling, or bands of subcutaneous tissue extending into the dermis can be assessed through sonography to ensure adequate surveillance of the progress of such therapies [12]. 

Last but not least, the integration of US imaging techniques facilitates real-time assessment of polydioxanone (PDO) threads, which serve as biocompatible synthetic polymers for non-surgical facial rejuvenation and tissue enhancement [15]. 

In this review article, we aimed to summarize the role of ultrasonography in the personalization of aesthetic procedures with hyaluronic acid, bio stimulators, and other types of widely popular injectables and the reasons why we should include high-frequency US in our daily practice, especially for handling possible related complications. As different types of complications exist (immediate, early, late, or delayed), their quick diagnosis is essential, and ultrasonography may prove to be an ideal tool for this purpose.

## 2. Ultrasound Characterization of Cosmetic Fillers: Integrating Schematic Illustrations and Ultrasound Imaging

In the context of aesthetic dermatology, conducting a comprehensive US examination of the entire facial area and submandibular regions is essential to provide in-depth anatomical data for effective patient management [16]. Dermatologic ultrasound examinations necessitate specialized equipment and operator expertise [16]. The ideal ultrasound machine for this purpose should be equipped with a color Doppler function and a compact linear multifrequency probe or a linear probe with a frequency range of at least 15 MHz [17]. Frequency selection is a critical consideration in dermatologic ultrasound [17]. Higher frequencies yield superior image resolution and the ability to assess finer details of skin structures [17]. For monitoring epidermal lesions, a transducer with a frequency range of 75–100 MHz is recommended [17]. However, when imaging encompasses the dermis and subcutaneous tissue, a transducer with a frequency range of 20–30 MHz is more suitable [18].

Typically, ultrasound machines within the 15–46 MHz frequency range can detect subcutaneous vascularization but may not reveal dermal vessels. However, utilizing a 70 MHz frequency allows for the observation of lower dermal vessels in specific regions, which often exhibit low velocities, typically measuring 15 cm/s or less [16]. The ‘hockey stick’ transducer is a versatile multifrequency probe, capable of reaching up to 13 MHz, with a hockey stick-shaped design that enhances sensitivity to both Doppler and color flow [16]. Its configuration ensures full skin-surface contact, minimizing scattering artifacts, making it an excellent choice for imaging small superficial structures, especially for vascular images [16].

Notable examples of commonly employed dedicated skin ultrasound systems include DermaScan C (“Cortex Technology, Hadsund, Denmark”), DUB-USB (“Taberna Pro Medicum, Lüneburg, Germany”), Episcan I-200 (“Longport, Inc., Silchester, Great Britain”), and DermaMed (“Dramiński S.A., Olsztyn, Poland”) [18].

**Figure 1:**
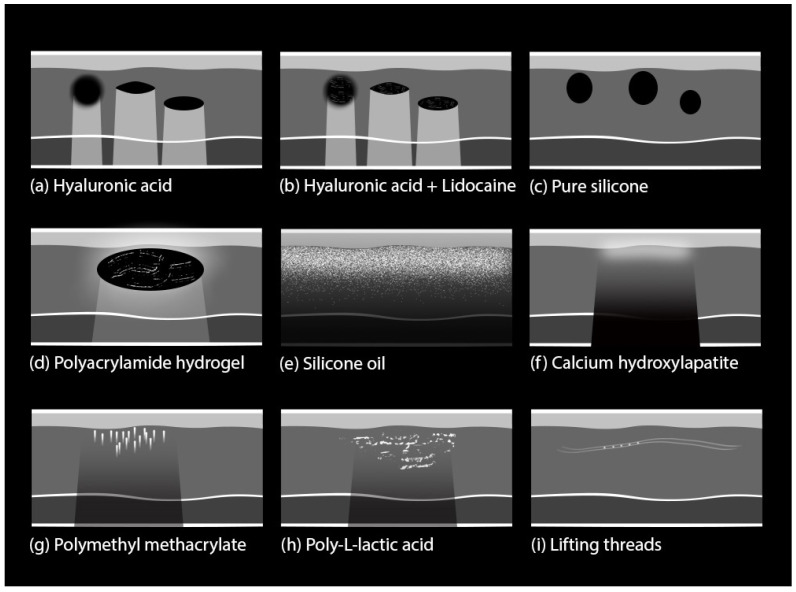
Schematic overview of key characteristics in common cosmetic fillers and lifting threads. The illustration also shows different artifact patterns: (**a**,**b**) strong posterior acoustic enhancement or reinforcement; (**c**) no visible artifacts, due to low difference between the acoustic impedance of pure silicone and surrounding tissue; (**d**) slight posterior acoustic reinforcement; (**e**) diffuse reverberation artifacts known as ‘snowstorm’ pattern; (**f**) posterior acoustic shadowing, due to calcium; (**g**) focal reverberation artifacts known as ‘mini-comet tail’; (**h**) posterior acoustic shadowing and (**i**) absorbable threads do not produce posterior acoustic shadowing.

This chapter will cover in detail the sonographic features of common biological (degradable) and synthetic (non-degradable) aesthetic fillers. The primary attributes of the most prevalent cosmetic fillers are visually encapsulated in Figure 1, providing a schematic overview. In the following sections, we will provide a thorough analysis of the ultrasound characteristics specific to each filler type. 

Hyaluronic acid (HA): HA is the most commonly used biodegradable filler, and it can be found in formulations that are either pure or combined with co-agents such as lidocaine. The pure formulations are visible on sonography as well-defined spherical, anechoic structures, that form a “pseudocystic” appearance [19]. The formulations with pre-incorporated lidocaine possess similar characteristics but typically exhibit interior linear echoes within the subcutaneous pseudocysts [19]. 

**Figure 2:**
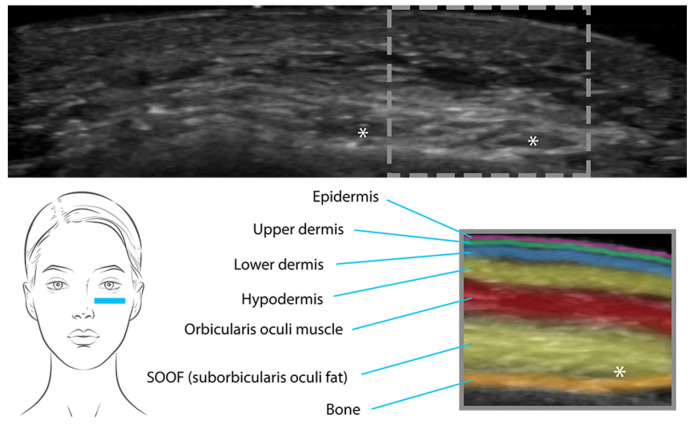
Pseudocystic structures (*) of high-density HA placed near the periosteum in the tear- through area.

They are usually injected at various depths at the subcutaneous level, based on the specific aesthetic goals, the treated area, and the density of the filler material. The density of a filler refers to its thickness or viscosity. High-density pseudocystic structures are usually small- to medium-sized and are typically placed in the deep hypodermis or near the periosteum [20]. Low-density HA structures are smaller in size and are placed more superficially in the hypodermis [20]. Different filler materials have varying degrees of density, a characteristic that plays a role in determining whether they are placed deeply or superficially [20]. After several months, the aspect may change from anechoic to hypoechoic as the filler loses its content in water and the pseudocysts gradually decrease in size as they are naturally dissolved [7] (Figure 2).

Poly-L-lactic acid (PLLA): injectable microparticles of PLLA represent a unique type of dermal filler that stimulates fibroblasts, the key cells involved in collagen production [21].

**Figure 3:**
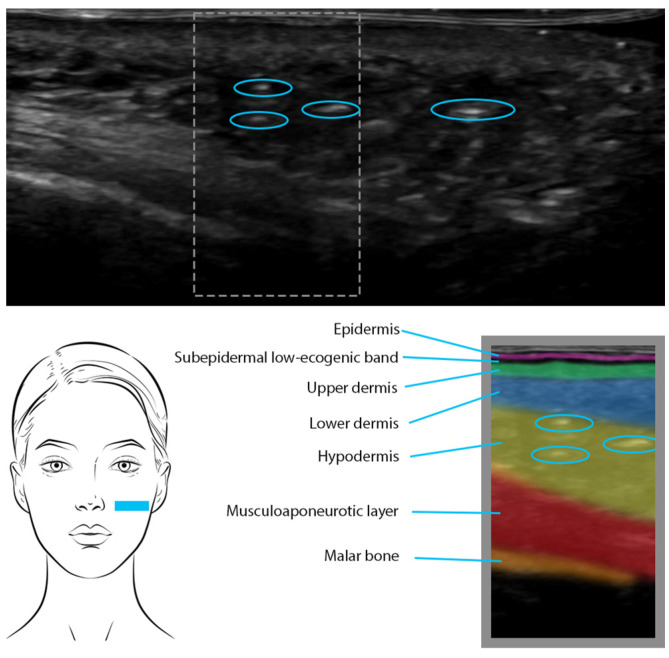
PLLA was injected 7 months ago in the cheeks. US shows hyperechoic patches (blue circles) in the lower dermis and hypodermis, causing a diffuse mottled appearance.

PLLA is usually injected into the reticular dermis or hypodermis. Unlike traditional dermal fillers, PLLA is not retained within the tissues as a deposit. It causes a controlled and localized induction of fibrosis, as part of the natural collagen-stimulating process that ultimately contributes to skin rejuvenation. The fibrotic tissue response is visible on US as hyperechoic patches with posterior shadowing that create a mottled appearance (Figure 3). Overall, the injected areas usually exhibit a similar echogenicity as the surrounding tissue, and the detection of the mottled pattern makes the presence and distribution of the filler distinguishable during US examinations [22].

Calcium Hydroxyapatite (CaHA): CaHA is also a collagen-stimulating filler composed of CaHA microspheres suspended in an aqueous gel carrier, allowing a smooth and precise delivery into the dermal or subdermal layers [23].

On US imaging, CaHA appears as a hyperechoic linear and undulating band. Notably, the appearance of CaHA on US is often accompanied by variable degrees of posterior acoustic shadowing, where sound waves are attenuated by the dense material, resulting in a darker region behind the filler deposit [8,23].

Polymethyl methacrylate (PMMA): PMMA fillers comprise polymethyl methacrylate microspheres suspended in a biocompatible collagen carrier that work to create a scaffold-like structure beneath the skin and to stimulate collagen production over time [24]. By reflecting US waves, these microspheres give rise to hyperechoic mass-like structures that generate posterior tiny, bright linear or V-shaped trailing reverberance, and are thus called comet-tail artifacts [8].

Polyacrylamide hydrogel (PAAG): PAAG is a synthetic hydrogel composed of acrylamide monomers that crosslink to form a three-dimensional network [25]. US imaging allows for the visualization of PAAG deposits, which appear as anechoic oval or round-shaped structures, with a homogenous texture, that produce posterior enhancement [26]. In contrast to HA, PAAG tends to maintain its size, echogenicity, and shape over long periods.

Silicone: Silicone is a prevalent substance employed for various aesthetic enhancement procedures. It exists in two distinct forms—pure silicone and silicone oil—each presenting unique characteristics on US imaging.

On sonography, pure silicone appears as an anechoic oval aspect characterized by a region without echoes, resembling a smooth, oval-shaped structure [27]. Notably, the shape and size of this anechoic oval remain consistent over time, adding a degree of stability to its diagnostic identification [27].

**Figure 4:**
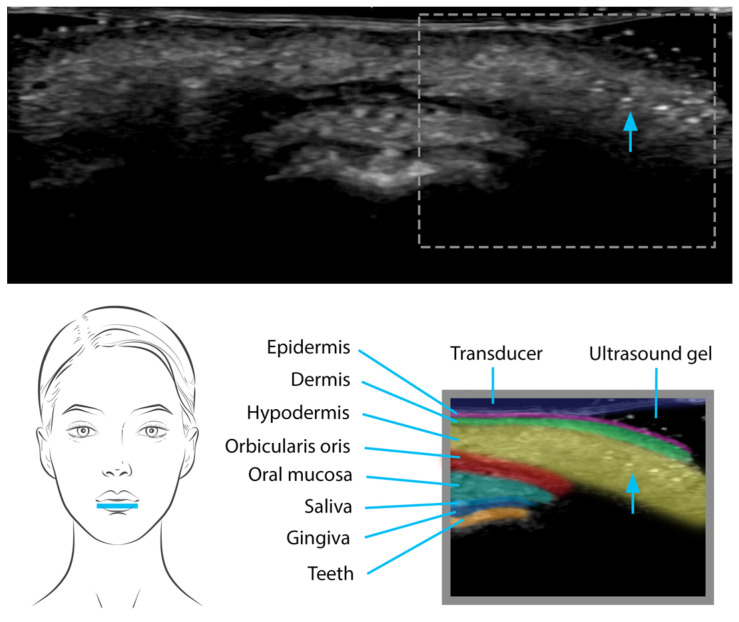
Silicone oil injection in the lower lip seen on US as well-defined, hyperechoic mass-like deposits that produce diffuse posterior reverberation (“snow storm pattern”).

In contrast, the US appearance of silicone oil differs significantly. Silicone oil manifests as a hyperechoic extended deposit, forming a distinct layer that is injected just beneath the dermis. This positioning gives rise to a particular posterior acoustic reverberation artifact characterized by a “snowstorm” pattern—a blurred, turbulent whitish dispersion on the US image [28] (Figure 4). This pattern arises due to multiple sound-wave reflections caused by the interaction between the silicone oil layer and the surrounding tissues. The distinct “snowstorm” pattern associated with silicone oil enables practitioners to track its location and potential migration over time [27].

### 2.1. Advancing Filler Complication Management with the Aid of Ultrasound

This section aims to explore the pivotal role of US imaging in advancing the management of complications associated with soft-tissue filler injections, from early detection and characterization to procedure guidance and post-treatment monitoring.

By offering real-time visualization and assessment of blood flow patterns, anatomical structures, and tissue alterations, US empowers clinicians to accurately diagnose complications such as vascular compromise, filler migration, the development of nodules and granulomas, hypersensitivity reactions, infections, and even dermopathies.

#### 2.1.1. Vascular Compromise

Soft-tissue ischemia or necrosis is a rare yet potentially dramatic complication associated with aesthetic fillers. Vascular compromise can occur following external vascular compression (arterial or venous, although it is more commonly associated with arterial compression) due to a significant amount of filler injected into a compacted area [29,30]. It can also occur following intravascular occlusion due to the intra-arterial injection of filler material with subsequent embolization in progressively smaller vessels [29,30]. Regardless of the etiology, further characteristics of fillers can have an impact on the degree of obstruction and on the possibility of response to treatment, such as filler particular composition, the amount of injected material, and the targeted anatomical zone [31]. Accordingly, due to the complex vascular anatomy, areas such as the nose and glabella should be approached with extreme caution [11].

For the glabella region, the transducer should be positioned axially, centered on the glabella, and extending to the superior orbital rim. From that point, sliding movements in the medial, lateral, and cranial directions should be performed for optimal imaging.

It is crucial to be aware of two significant vascular structures: the supraorbital artery and the supratrochlear artery, which originate from the ophthalmic artery. Thus, the intravascular injection of fillers can result in blindness. Although it is commonly recognized that these vessels typically exhibit greater depth in the lower forehead and are relatively more superficial in the upper forehead, US can offer a more precise assessment of potential variations in vessel depth and positioning, allowing for an adaptation of the injection plane if necessary [32,33,34].

For the nose evaluation, it is recommended to position the transducer sagittally, spanning from the glabella to the nasal tip. To ensure optimal contact, it is advisable to apply a generous amount of ultrasound gel on the probe and to perform sliding motions in both upward and downward directions, as well as lateral ones along the nose. The nasal bone and cartilages should be used as reference points. US imaging facilitates the visualization of the essential vascular structures, which include the dorsal nasal artery, the intercanthal vein, and the external nasal artery. Due to the small diameter of the nasal vessels, it is essential to avoid applying any pressure with the probe [11].

Duplex US is a valuable source of information regarding vascular variability [29]. It provides the possibility of vascular mapping before and after volumetric procedures, and has a high level of diagnostic precision in assessing the blood flow changes and arterial pulsations. Based on diameter and pressure measurements, injured vessels can be identified and additional measures can be conducted [35]. If hyaluronic acid is used, guided hyaluronidase placement directly into the obstructed vessel can be performed [36].

#### 2.1.2. Filler Migration

Filler migration refers to the displacement of filler material away from the targeted area of injection. Several mechanisms were proposed for the pathogenesis of filler migration: inadequate technique, muscle activity, gravitational forces, pressure-induced rearrangement, or high amount of filler placed into a small, restricted area [37]. Regardless of the etiology, filler migration can result in palpable masses or swellings that can be aesthetically unpleasant. Furthermore, if soft-tissue substances migrate to sensitive areas, the results can be debilitating [38]. The glabella, eyelids, and forehead are the most typical sites for filler migration, given their distinctive anatomical features [39]. High-frequency US can be used to prevent this complication. Guided placement of smaller droplets of volumetric compounds and mapping the area before injection can reduce the chance of filler displacement [40]. Moreover, US has a peculiar role in both the diagnosis and treatment of filler migration, being able to precisely track changes in filler position over time and provide assisted injection of hyaluronidase if needed [41].

#### 2.1.3. Localized Tissue Reactions: Non-Inflammatory Nodules and Granulomas

Non-inflammatory nodules and granulomas are distinct entities that manifest as localized tissue reactions. Although both conditions arise following filler injections, they differ in pathophysiology, histological features, clinical presentation, and therapeutic approach.

Non-inflammatory nodules: the development of nodules or lumps at the injection site is one of the most common complications of filler injections. These lesions are characterized by localized palpable masses that develop beneath the skin, at the site of filler injection [42]. They are primarily a result of overfilling, following improper technique placement or uneven distribution within the subcutaneous tissue [42]. Such nodules appear shortly after filler injection and hold characteristic features on Duplex-US: a specific pattern of a well-defined mass with no increase in vascularization [43]. Histologically, non-inflammatory nodules exhibit minimal inflammation and lack the classic granulomatous inflammatory response. Instead, they are composed of relatively homogeneous filler material with limited cellular infiltration [44]. Management typically involves conservative measures, such as observation, massage, and, in some cases, hyaluronidase injection in the case of hyaluronic acid-based fillers. US can help determining the appropriate depth and guiding massage technique or hyaluronidase injection, ensuring an effective and targeted management [44].

Granulomas: although non-inflammatory nodules appear due to mechanical factors associated with filler placement, granulomas result from a complex interplay between the immune reaction and the foreign-body response to the injected material. With a rather low estimated incidence, foreign-body granulomas can appear within a latent period of months or years after soft-tissue augmentation [45]. They are composed of aggregates of immune cells, notably macrophages, forming multinucleated giant cells surrounded by lymphocytes and fibroblasts. Histologically, granulomas showcase the hallmark granulomatous pattern, reflecting a delayed-type hypersensitivity reaction [46]. Several factors may influence the development of these reactions: increased injected volumes, use of non-degradable fillers, and previous trauma at the injection site [42]. Unlike non-inflammatory nodules, the ultrasonographic correspondent of foreign-body granulomas is that of a highly vascularized, poorly defined mass with a non-homogenous pattern [47]. Additionally, US can be very helpful in determining the best course of action; if an encapsulated granuloma is present, surgical excision is required, whereas in other cases, guided hyaluronidase and intralesional corticosteroids injections should be administered [48].

US also serves as a valuable tool for post-treatment monitoring and follow-up. Serial US assessments enable clinicians to track the resolution of complications over time, providing insights into the effectiveness of therapeutic interventions. This dynamic monitoring approach allows further adjustments in management plans, based on the evolving characteristics of nodules or granulomas [49]. Furthermore, US aids in identifying potential recurrences or the development of new complications, enabling early intervention and minimizing patient discomfort.

#### 2.1.4. Hypersensitivity Reactions

Some patients may experience hypersensitivity reactions to soft-tissue fillers, as these substances are fundamentally considered foreign bodies [42]. Products with animal-derived components such as bovine collagen are more prone to induce this kind of reactions due to increased immunogenicity [50]. Whether they have an early (the first hours after the procedure) or delayed onsets (days to weeks), the clinical presentation is quite similar. The most common manifestations include edema, pruritus, and erythema [51]. However nodules and granulomas might also appear as delayed-type responses [51]. US outlines the imagistic attributes of filler-associated edema, helping the clinician to identify this intricated reaction [43]. On US, the distinctive depiction of swelling is represented by areas of augmented echogenicity and increased thickness of surrounding tissue [52].

#### 2.1.5. Infections

Although relatively uncommon, infections following volumetric procedures may develop due to skin breakage at the injection site [53]. Abscesses presenting as painful fluctuating nodules are usually an indicator of early bacterial infection [54]. These entities can be easily identified with Duplex-US as uni- or multiloculated fluid collections with enhanced vascularity in the surrounding area [43]. Furthermore, if needed, US can provide assisted incision and drainage [54]. Some late-onset bacterial infections have been correlated with biofilm development [55]. Biofilm can appear when complex collections of the skin’s surface bacteria attach to the injected filler and secrete a protective, nourishing, and adhesive matrix [42]. Regardless of its clinical presentation as nodules, abscesses, or granulomas, once a biofilm is suspected, US-guided interventions facilitate accurate biofilm debridement, aspiration of fluid collections, targeted delivery of therapeutic agents, and even filler removal if necessary [55].

#### 2.1.6. Dermatopathies

Dermatopathies can manifest as late-onset adverse reactions following filler injection. Clinical diagnosis can be challenging given the similarities between the associated dermatopathies and prevalent dermatologic conditions (angioedema and morphea) [26]. Permanent fillers, like liquid silicone, are more prone to elicit severe dermal fibrotic reactions at the injection site [56]. In time, actual subcutaneous fibrotic masses with retraction and limited mobility of the overlying skin can develop, mimicking morphea-like lesions [38]. The utility of high-frequency US stands in establishing the pathogenic causality between the two. US provides crucial information not only about dermal thickness and echogenicity but also about the filler’s type, its precise location, and consequences on the surrounding tissue [57]. If filler deposits confine exactly to the limits of the cutaneous injury, the clinical correlation between the filler and morphea-like lesions can be prompted [43].

### 2.2. The Application of Ultrasound Technology in Non-Surgical Aesthetic Procedures

#### 2.2.1. Mesotherapy

Nowadays, minimally invasive aesthetic treatments have achieved widespread prevalence on an international scale [15,16,58,59,60]. US emerges as a valuable tool in monitoring intradermal mesotherapy and assessing its clinical effectiveness and possible adverse reactions [13].

Mesotherapy predominantly aims to revitalize the skin by using various subcutaneous or intradermal injections, allowing the introduction of an extensive selection of biocompatible compounds: multivitamins, minerals, caffeine, nutrients, homeopathic agents (artichoke, melilotus, ginkgo biloba), hormones, proteins, enzymes, (hyaluronidase, collagenase), lipolytic agents (phosphatidylcholine, deoxycholic acid), hyaluronic acid, amino acids (carnitine), pentoxifylline, coumarin, calcium pyruvate, and aminophylline [14,16]. These bioactive substances are usually used for non-invasive cutaneous biorejuvenation purposes or treatment of alopecia and cellulitis [16].

Mesotherapy increases the blood and lymphatic flow in the dermal layer, resulting in the restoration of skin texture and the prevention of skin aging [14]. Age-related changes in the dermal layer can be assessed using high-frequency US [14]. Moreover, the existence and extent of the subepidermal low-echogenic band (SLEB) on the high-frequency US are linked to photoaging: as the echogenicity of the SLEB decreases, the severity of photoaging increases [14].

Mesotherapy can lead to inflammation in the dermal and/or hypodermal layers, with a higher incidence in the hypodermis, resulting in lobular or mixed panniculitis [16]. Consequently, a decrease in echogenicity within the dermis and an increase in echogenicity within the hypodermis can be identified [16].

Occasionally, a granulomatous response can be encountered as well [2,16,61]. Within these cases, the presence of hypoechoic tissue and nodules/pseudo nodules becomes observable [2,16,61]. In certain situations, regional lymphedema signs might be seen, including epidermal, dermal, and hypodermal thickening alongside the echogenicity changes within these layers [2,16,61]. Sporadically, abscesses or fluid collections might be encountered at the injection areas [2,16,61]. Color Doppler imaging may reveal increased vascularity within the affected skin layers [2,16,61].

#### 2.2.2. Radiofrequency

Radiofrequency (RF) energy is an electric current, with a high frequency, that has been used for several decades for tissue electrodessication, endovenous ablation, and cardiac catheter ablation [62]. Frequencies in the range of 200 kHz to 6 MHz are most commonly used in medical aesthetic fields [62]. When RF is transmitted to skin tissue, molecules in the skin create friction and generate heat at 40–60 °C, causing structural change and the denaturation or coagulation of proteins and collagen [62]. Thus it stimulates wound healing and promotes the production of new collagen [62]. In cosmetic dermatology, RF is considered a safe and efficient procedure and it is primarily used for non-invasive skin rejuvenation, tightening, scar treatment, body contouring, and the reduction of cellulite [62].

In facial rejuvenation and skin laxity, RF serves as a non-invasive approach, often targeting deeper layers without the ablation of the epidermis and dermis [16,62]. RF thermal stimulation through unipolar, monopolar, or bipolar devices induces a minor inflammatory response within fibroblasts, triggering neocollagenesis, neoelastogenesis, and other compounds that contribute to the enhancement of dermal structure [62].

US examination reveals thickening of the dermal and hypodermal layers, as well as reduced echogenicity within the dermis and increased echogenicity of the hypodermis [16]. Occasionally, thickening of the hypodermal septa may also be observed [16]. Color Doppler examination shows either hypovascularity or hypervascularity [2,16,61].

#### 2.2.3. Polydioxanone Tensor Threads

Tensor threads are used to treat skin sagging and to promote both collagen production and fibrosis [16]. In modern times, the prevalent choice leans towards the utilization of absorbable non-barbed variants, particularly those made of PDO, a material commonly found in sutures [16,63,64,65,66,67].

Frequently, PDO tensor threads are introduced into the hypodermis [16]. Yet, there are instances where they might be located in the dermal layer, which often leads to complications [16]. Under US examination, they appear as hyperechoic structures with bilaminar or trilaminar configurations, occasionally producing a mild posterior acoustic shadowing artifact [16].

Nevertheless, absorbable threads undergo fragmentation within a span of 2 to 3 months, resulting in a loss of tension [16]. When these fragmented threads are situated close to or within the dermal layer, one may observe hypoechoic tissue characterized by granulomatous inflammatory reactions surrounding those components [16].

Color Doppler US may reveal variable vascularity depending on the extent of inflammation surrounding the threads [23].

#### 2.2.4. Autologous Fat Transfer

It is also known as autologous fat grafting, lipotransfer, liposculpting, or lipofilling and it is based on fat injection for soft-tissue augmentation and thus facial rejuvenation [16,68,69,70,71].

Incorporating duplex US in our daily practice can provide valuable insights during a lipofilling procedure, making its utilization an essential aspect to ensure a secure lipofilling treatment [68]. As such, US assessment serves as a significant tool for enhancing the effectiveness and safety of lipofilling procedures, particularly in regions with increased vascularity and elevated risk [68].

Fat grafts appear on US images as round or oval, well-defined heterogenous hypoechoic masses, often accompanied by hyperechoic linear septae [2,16,61,68]. They disturb the regular tissue arrangement and do not align with the anatomical orientations of the cutaneous layers [2,16,61,68]. They are typically situated in the hypodermis, but they can also be present within facial muscles, notably in areas like the orbicularis oculi muscles, around the eyelids or the orbicularis oris muscle, and surrounding the lips [2,16,61,68]. Moreover, the use of Doppler US imaging could enhance both the effectiveness and safety of liposuction and lipofilling, as well as also assisting in postoperative care [68].

Doppler US can assist medical practitioners in addressing aesthetic areas that are susceptible to uneven contours [68]. This includes identifying the appropriate suction plane, thus reducing the chances of irregularities [68]. Regarding facial autologous fat injections, where variations in facial vasculature are common, Doppler US plays a role in preoperative vascular mapping and identifying the existence of (permanent) filler deposits from prior treatments [68]. Fat grafts appear hypovascular on color Doppler US images [2,16,61,68].

#### 2.2.5. Implants

Implants are synthetic structures used for volume restoration and contour enhancement [16]. Multiple implant types are available (silicone gel, fat, saline, autologous cartilage, porous high-density polyethylene, or bone) and suitable for insertion into various anatomical sites like the nasal area, cheeks, chin, or body [2,16,23,61].

On US, silicone implants appear as well-defined anechoic structures, with an oval shape, displaying along their periphery a single-layer, double-layer, or triple-layer configuration [16]. Polyethylene and cartilage implants can be seen as well-defined bands, the former hyperechoic, whereas the latter is hypoechoic [16].

As implants are susceptible to rupture, US is a valuable tool to assess whether it is intracapsular or extracapsular [16]. Intracapsular rupture is defined by the presence of echoes within it, the emergence of wavy lines known as the “stepladder sign,” and the discontinuity of its edges [16]. The extracapsular rupture characteristics include hyperechoic deposits and the presence of the “snowstorm” sign, which manifests as a widespread acoustic reverberation towards the outer edges of the implant [16].

As different levels of inflammation might be encountered at the sites of the implants, the color Doppler usually detects varying levels of vascularization [16].

### 2.3. The Role of Ultrasonography in the Administration of Hyaluronidase

Hyaluronidase is an endo-N-acetyl hexosaminidase and functions as an endoglycosidase that cleaves hyaluronic acid glycosidic bonds, fragmenting it into monosaccharides and thus inducing its depolymerization [16,72].

Hyaluronidase has multiple roles encompassing the dissolution of hyaluronic acid fillers, the management of granulomatous reactions, and addressing necrosis linked to filler injections [72]. Consequently, off-label use of hyaluronidase injections has been documented as effective in treating both nodules and the Tyndall effect [41]. The administration of hyaluronidase into the skin is documented to exhibit immediate effects lasting for approximately 24 to 48 h, with a half-life of 2 min, and subsequent metabolic processing occurring in the liver and kidneys [41]. The typical dosage ranges from 5 to 75 units, and some researchers have proposed that dissolving a 0.1 mL injection of 20 mg/mL hyaluronic acid would require about 5 units of hyaluronidase [41].

The use of ultrasonography guidance for the hyaluronidase injection technique has several benefits [72]. US offers a quick, uncomplicated approach without any radiation exposure risk or discomfort for patients [72]. Precisely delivering the enzyme within the hyaluronic acid filler pocket appears to be fundamental for achieving successful outcomes, and clinically evident symptoms to resolve more promptly [16,41,71,72,73,74]. This targeted approach proves to be more efficacious than administering injections blindly [16,41,72]. Furthermore, the application of US-guided treatment allows the reduction of the amount of hyaluronidase required [16,41,72]. The use of US imagery enables the avoidance of critical blood vessels and nerves [72]. In cases of severe adverse events like blindness or necrosis, this method precisely aids in preventing their exacerbation [72]. This approach effectively helps in the filler’s dissolution due to the accurate pre-definition of the targeted area [72].

## 3. Ultrasound Versus Alternative Imaging Modalities

The implementation of imaging modalities dedicated to ensuring safety within the aesthetic domain is of paramount importance to attain the best possible patient outcomes. Choosing the most appropriate imaging technique for cosmetic filler procedures depends on the specific clinical scenario and objectives.

Ultrasound is a non-invasive and easily accessible tool that employs high-frequency sound waves to create real-time imaging. It provides immediate feedback during procedures, allowing practitioners to dynamically monitor filler placement [75].

A very important aspect is its high axial spatial resolution, which allows for detailed imaging of superficial structures, hardly visualized with Magnetic resonance imaging (MRI) or computed tomography (CT) [76].

Furthermore, ultrasound does not expose patients to ionizing radiation, making it safe for repeated evaluations. There is no requirement for contrast medium injection, compared to MRI and CT. The contrast mediums used in these procedures can have significant biological effects and potentially lead to adverse reactions, including cutaneous nephrogenic fibrosis and kidney disease [76]. Also, patients with pacemakers and metallic prostheses can safely undergo US examinations without magnetic field exposure [76].

A significant limitation of US pertains to the proficiency of the operator. Training in ultrasound utilization and interpretation is essential, and continuous practice is imperative. The DERMUS group recommends that a minimum of 300 ultrasound procedures be performed annually to attain a baseline level of competency. Likewise, it is highly recommended that the dermatologist serve as both the practitioner and the sonographer due to their extensive training in dermatological pathologies and their capability to correlate ultrasound findings with the clinical manifestation of cutaneous lesions [17].

Another important constraint is that US-based evaluations can be susceptible to observer bias and may exhibit reduced reproducibility when conducted by a different investigator.

In the present paper, the ultrasound assessments were conducted collaboratively by the same two investigators: a dermatologist who had received specialized training in ultrasound techniques and aesthetic filler applications, and a proficient sonographer highly skilled in soft-tissue ultrasound examinations. All measurements were conducted using the same device, a LOGIQ E9 XD Clear machine (GE Healthcare, Milwaukee, WI), with high-resolution linear transducers of 16 MHZ. This approach, although intended to ensure consistency, may introduce potential bias due to a lack of multiple perspectives or ultrasound equipment.

The application of this imaging modality, particularly in the context of fillers, continues to necessitate additional patient-oriented studies to facilitate its complete integration into daily medical practice. Similar challenges are encountered in other medical specialties, and there remains a need for extensive research to assess its sensitivity and specificity [77,78].

MRI provides excellent soft-tissue contrast and is valuable for assessing deeper structures [79]. It is particularly useful when evaluating the effects of cosmetic procedures on underlying tissues. Due to multiplanar acquisitions and precise depiction of anatomical landmarks, MRI is considered a valuable diagnostic modality that facilitates the evaluation of filler localization and surrounding tissue changes [80]. MRI not only has an excellent ability to distinguish between potential filler complications (granulomatous reactions, fibrosis, abscesses) but sometimes, due to better spatial resolution, is preferred over high-frequency US when it comes to filler misplacement [38,81]. However, its value in the clinical practice is quite limited due to its inability to precisely establish the type of the injected filler. As most volumetric compounds, with the exception of silicones, have similar water content, they depict nonspecific patterns on MRI [79]. Additionally, MRI is not suitable for real-time monitoring during procedures due to its longer acquisition times.

CT scans offer high-resolution imaging and are well-suited for assessing bone structures and deeper tissues. They can be beneficial when assessing the long-term effects of cosmetic procedures, especially those involving bony structures or implant placement. One particular feature is its capacity to identify calcifications, which can be a hallmark for a precise type of filler or associated complications [79]. However, CT is not an optimal imaging technique to evaluate cosmetic fillers due to the patient’s extensive exposure to ionizing radiation [82]. Positron emission tomography is also inadequate for filler assessment. Most volumetric substances possess a physiologic, high absorption of fluorodeoxyglucose (FDG) which may lead to a diagnostic pitfall. Usually, FDG uptake is associated with infection or inflammation, but in this case, increased metabolic activity can be seen in patients with or without filler-related complications [38].

## 4. Conclusions

Ultrasonography is a noninvasive, dynamic, and cost-effective imaging technique that allows for a comprehensive targeted assessment of aesthetic procedures. It offers precise visualization of skin layers, enabling real-time recognition of prominent cosmetic fillers, biostimulators, and tensor threads. This method supplies valuable insights into skin depth, infused volume, and potential complications associated with exogenous products injected into the human body. It also gives valuable insights into non-surgical aesthetic procedures and into the administration of hyaluronidase, which may have further implications in the clinical practice.

Given these remarkable advantages and compared to other alternative imaging modalities such as MRI and CT, it is our belief that high-frequency US should no longer be viewed as an optional tool but rather as an indispensable one for personalized aesthetic procedures based on the evidence presented in the article. US is not only an imaging modality but rather a comprehensive clinical, physical, and imaging assessment. Its integration into the training and practice of dermatologists, facial plastic surgeons, ENT specialists, and other relevant medical professionals is not only recommended but should be actively encouraged for a more precise, safe, and effective medical approach.

## Data Availability

This review summarizes data reported in the literature and it does not report primary data.

## Abbreviations

The following abbreviations are used in this manuscript:

CaHA	Calcium Hydroxyapatite
ENT	Ear, nose, and throat
FDG	Fluorodeoxyglucose
HA	Hyaluronic acid
MHz	Megahertz
PAAG	Polyacrylamide hydrogel
PDO	Polydioxanone
PLLA	Poly-L-lactic acid
PMMA	Polymethyl methacrylate
RF	Radiofrequency
SLEB	Subepidermal low-echogenic band
US	Ultrasound
